# Beyond the Microbiota: Understanding the Role of the Enteric Nervous System in Parkinson’s Disease from Mice to Human

**DOI:** 10.3390/biomedicines11061560

**Published:** 2023-05-27

**Authors:** Martina Montanari, Paola Imbriani, Paola Bonsi, Giuseppina Martella, Antonella Peppe

**Affiliations:** 1 Laboratory of Neurophysiology and Plasticity, IRCCS Fondazione Santa Lucia, 00143 Rome, Italy; martina.montanari@students.uniroma2.eu (M.M.); p.imbriani@hsantalucia.it (P.I.); p.bonsi@hsantalucia.it (P.B.); 2 Department of Systems Neuroscience, University of Rome Tor Vergata, 00133 Rome, Italy; 3 Clinical Neuroscience, IRCCS Fondazione Santa Lucia, 00179 Rome, Italy

**Keywords:** Parkinson’s disease, gut–brain axis, enteric nervous system, central nervous system, neurons, glia cells, non-motor symptoms, gastrointestinal dysfunction, microbiota, rodent models, clinical evidence

## Abstract

The enteric nervous system (ENS) is a nerve network composed of neurons and glial cells that regulates the motor and secretory functions of the gastrointestinal (GI) tract. There is abundant evidence of mutual communication between the brain and the GI tract. Dysfunction of these connections appears to be involved in the pathophysiology of Parkinson’s disease (PD). Alterations in the ENS have been shown to occur very early in PD, even before central nervous system (CNS) involvement. Post-mortem studies of PD patients have shown aggregation of α-synuclein (αS) in specific subtypes of neurons in the ENS. Subsequently, αS spreads retrogradely in the CNS through preganglionic vagal fibers to this nerve’s dorsal motor nucleus (DMV) and other central nervous structures. Here, we highlight the role of the ENS in PD pathogenesis based on evidence observed in animal models and using a translational perspective. While acknowledging the putative role of the microbiome in the gut–brain axis (GBA), this review provides a comprehensive view of the ENS not only as a “second brain”, but also as a window into the “first brain”, a potentially crucial element in the search for new therapeutic approaches that can delay and even cure the disease.

## 1. Introduction

The gastrointestinal (GI) tract is a long tubular structure that harbors highly diverse and complex communities of microorganisms, including bacteria, archaea, microeukaryotes, and viruses that readily vary with diet, pharmacological intervention, and disease [1].

Numerous articles have described differences in the composition and function of the gut microbiome of healthy individuals and patients of metabolic, autoimmune, and neurodegenerative diseases [2,3,4]. In the pathogenesis of brain disorders, the possible involvement of peripheral organs has always been marginal. However, it is now well established that the environment of the GI tract and distant organs, such as the brain, is affected by the homeostasis of the gut microbiota and the host’s health [4,5,6]. Early colonization of the gut microbiota is vital for brain function and behavior, considering that its absence results in impairment of the blood–brain barrier [7].

The enteric nervous system (ENS), the part of the nervous system closest to the microbiome, has recently become the subject of in-depth investigations [5,8,9]. It is now known that the microbiome affects the development and functioning of the ENS, modulating it throughout life [4]. Since a wide range of neuropathies are associated with ENS dysfunction, we believe it is worth taking a closer look at it [5,8,9].

The ENS is derived from pre-enteric (rhombencephalon) and sacral neural crest cells and includes efferent and afferent neurons, interneurons, and glial cells.

This well-organized and integrated network of plexuses is relatively independent because it can control gut function independently of CNS sympathetic and parasympathetic innervations [10,11]. However, the ENS is not autonomous, since several CNS structures monitor and regulate what is happening in the GI tract through biochemical signals [12,13]. The gut–brain axis (GBA) consists of a bidirectional communication between the CNS and ENS, linking the emotional and cognitive centers of the brain with peripheral gut functions [14,15,16]. The GBA connects CNS cognitive centers with gut centers, regulating immune activation, enteric reflex, entero-endocrine signaling, and intestinal permeability [5,17,18]. The bidirectional communication between the gut and the brain implies a vital role for the gut microbiome through regulating host metabolism and immune and vascular systems [19]. In addition, the gut microbiome can also influence the CNS through the vagus nerve by transmitting signals from the gut microbiome to the brain and vice versa in both health and disease through neuro-immuno-endocrine mediators [17,18,20,21]. 

Disruption of GBA results in alterations in intestinal motility and secretion causes visceral hypersensitivity and leads to cellular changes in the entero-endocrine and immune systems [20]. 

Considering this complexity, the GI tract can be affected by aging, irritable bowel syndrome, severe inflammatory conditions (Crohn’s disease and ulcerative colitis), and even neurodegenerative diseases such as Parkinson’s and Alzheimer’s [19,21,22,23,24].

Parkinson’s disease (PD) is a neurodegenerative disease characterized by the loss of dopaminergic cells in the Substantia Nigra pars compacta (SNpc) and brain accumulation of Lewy bodies (LB), which are abnormal aggregates of α-synuclein (αS) [25,26,27]. PD results from a synergistic interaction between genetic factors and environmental stressors in most patients, a condition termed “double-strike theory” [25,26,28,29].

Therefore, exploring the potential interaction between distinct genetic and environmental factors is essential to identify convergent pathways and potential molecular targets for neuroprotection [30]. PD patients are a heterogeneous group, varying in the age of disease onset, speed of progression, the severity of motor and non-motor symptoms, and the extent of central and peripheral inflammation [31,32,33,34,35,36]. Indeed, PD is characterized by motor features and numerous non-motor symptoms that include sensory abnormalities, fatigue, sleep disturbances, autonomic dysfunction, psychiatric disorders (depression, anxiety, and apathy), and others [32,36,37,38]. Orthostatic hypotension, urogenital system disorders, hypersalivation, swallowing impairment, delayed gastric emptying, and constipation are the common manifestations related to autonomic dysfunction in PD [39,40,41,42,43,44,45]. Constipation is one of the most frequent non-motor symptoms, affecting up to 80% of PD patients, and may precede the onset of motor symptoms by years [46,47,48,49,50]. In the premotor phase, idiopathic constipation is one of the most critical risk factors for the onset of PD and is associated with neurodegenerative changes in the ENS [12,51]. 

According to Braak’s classic hypothesis [52], neurodegenerative diseases, particularly PD, may recognize a peripheral origin when putative pathogens enter the mucosa of the GI tract, inducing misfolding and aggregation of the hallmark αS in specific subtypes of CNS neurons, then spreading retrogradely to the CNS via preganglionic vagal fibers to the dorsal motor nucleus (DMV) and, finally, to other central nerve structures [12,51,53,54].

Recently, two categories of PD patients have been identified: a brain-first (top-down) type, in which the αS pathology arises initially in the CNS and then in the peripheral autonomic nervous system, and a body-first (bottom-up) type, in which the pathology originates in the ENS and then spreads to the CNS [55].

As pointed out earlier, PD is now considered a systemic disorder despite its typical neurological manifestations. Several autonomic changes in peripheral organs have been described as symptoms and prodromal markers [43,56,57,58]. The GI tract is primarily affected, hence the importance of assessing early changes occurring in the ENS and interpreting their role in the pathogenesis of PD [43,57,58]. This could help to understand the relationship between α-synucleinopathy, inflammation, neuroprotection, and neurotoxicity, which characterize patients with PD [4,6,7,59].

Data from patients and animal models suggest that PD affects distinct subsets of neurons and glia in the ENS and that the latter may participate in the pathogenesis of this disorder [10,12,56]. Moreover, numerous publications have pointed out the highly complex gut–brain link in PD, laying the foundation for developing new biomarkers and therapies [60]. However, the microbiome appears strongly influenced by environment and socioeconomic background, thus presenting extreme heterogeneity among individuals and little uniqueness [60,61]. 

In this context, the present article aims to go beyond the microbiome and focus on the involvement of the ENS in PD, elucidating the interactions with the GBA [43,62]. Our goal is to expand knowledge on the pathophysiology of PD by paying particular attention to peripheral biomarkers within the ENS to identify new therapeutic strategies. Furthermore, because the manifestations of neuropathologies are parallel in the ENS and CNS, we believe that the ENS may represent a more accessible target for studies of neural function, histopathology, and biochemistry in PD [56,62]. We envision the ENS not only as a “second brain”, but also as a window into the “first brain”. 

## 2. Overview of the Enteric Nervous System: Anatomy and Function

**Figure 1:**
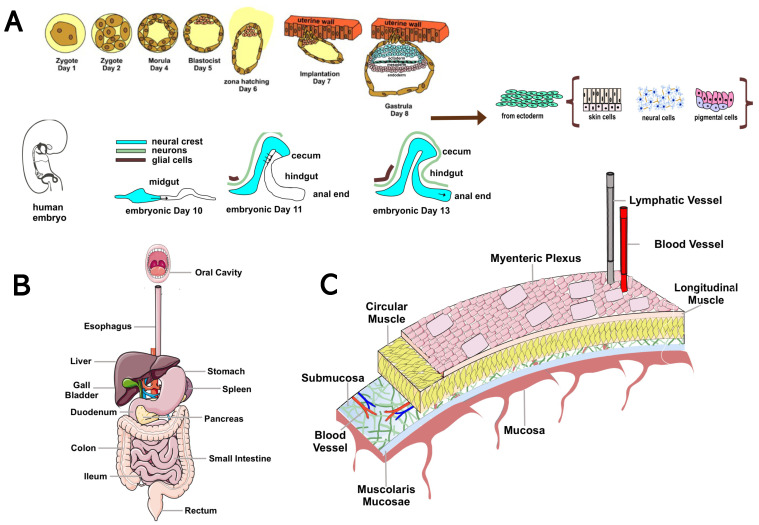
Overview of the anatomy and organization of the ENS. (**A**) Time course of ENS development. The ENS originates around the eighth day of embryonic life from neural crest progenitor cells (ENCDCs) with stem-like properties, which migrate through the GI tract and colonize it within five days. After invading the anterior intestine, these pre-ENCDCs migrate rostro-caudally, proliferating and differentiating into neurons and glia. During this process, the intestine elongates, changing shape from a straight line to a single curve, with the middle and small intestine closely adjacent. The cecal appendix grows and the entire intestine elongates further. At embryonic days 11 and 13, ENCDCs invade the colon by crossing the mesentery and transiting into the cecum. The cecal and trans mesenteric populations then fuse to form the ENS in the rostral colon. In humans, the ENS becomes functional in the last trimester of gestation and continues to develop after birth. (**B**) Schematic diagram of the human GI tract. (**C**) Organization of the ENS. NEs are organized into ganglionic plexuses: the myenteric plexus and the submucosal plexus. The ganglionic plexuses are enveloped by glial cells, such as CNS astrocytes, which form a proper blood–enteric barrier. The myenteric plexus is in the muscle tonaca between the layers of longitudinal and circular muscles. It consists of linear chains of numerous interconnected neurons that span the length of the GI and regulate its movements.

The ENS, the intrinsic innervation of the GI tract, is the largest and most complex division of vertebrates’ peripheral and autonomic nervous systems. In humans, the ENS contains 400–600 million neurons and an array of neurotransmitters and neuromodulators similar to those found in the CNS [11]. Unlike the CNS, in which efferent pathways are characterized by pre-ganglionic and post-ganglionic neurons [63], the axons of gut neurons in the ENS project to the sympathetic ganglia, brainstem, spinal cord, pancreas, gallbladder, and trachea [10]. The anatomy and physiology of the ENS have been studied since the 19th century, going so far as to demonstrate early in the last century how the peristaltic reflex (i.e., the pressure-induced propulsive activity of the intestines) is a local nervous mechanism that occurs in the absence of external nerve input [8]. Because of this autonomy and its complexity, Michael D. Gershon likened the ENS to a second brain [11]. Two-way communications between the ENS and the CNS are always active: the CNS can regulate or alter the normal functioning of the ENS and vice versa. For example, certain gut disorders impair the production of psychoactive substances such as serotonin (5-HT, 5-hydroxytryptamine), dopamine (DA), and opiates, which can affect mood [64]. Conversely, emotional states, such as intense anxiety, can cause colitis, constipation, irritable colon, or mucosal ulcers by stimulating peristalsis and hyperproduction of neurotransmitters [64]. The ENS originates around the eighth day of embryonic life from neural crest progenitor cells, endowed with stem-like properties, which migrate through the forming GI tract and colonize it within five days [11]. They subsequently differentiate into neurons and glia by integrating predetermined instructions with information from the microenvironment [9]. In humans, the ENS becomes functional in the last trimester of gestation and continues to develop after birth [9]. The ENS comprises small aggregations of nerve cells, the enteric ganglia, the neural connections between these ganglia, and the nerve fibers that supply effector tissues, including gut wall muscle, epithelial lining, intrinsic blood vessels, and gastroenteropancreatic endocrine cells [8,10,11,65]. Enteric neurons (NEs) are organized into ganglionic plexuses: the myenteric (Auerbach’s) plexus and the submucosal (Meissner’s) plexus. Ganglionic plexuses are enveloped by glial cells, such as CNS astrocytes, which form a proper blood–enteric barrier. Glial cells release enterocyte differentiation factors, participate in GI functions, and are involved in the pathogenesis of inflammatory disorders of the GI tract. Auerbach’s myenteric plexus, located in the muscle tonaca between the layers of longitudinal and circular muscles, consists of linear chains of numerous interconnected neurons that span the length of the GI tract and regulate its movements. Meissner’s submucosal plexus, located in the submucosa of the small and large intestines but absent in the esophagus and stomach, consists of ganglia stratified at different levels. It integrates sensory signals from the intestinal epithelium and contributes to the local control of secretion, intestinal absorption, blood flow, and submucosal muscle contraction [8,10,65] (Figure 1).

Twenty types of NEs characterized by different morphological, neurochemical, and electrophysiological aspects, connections, and functional roles have been identified [9,66,67]. Based on intracellular electrophysiological recordings, two types of NEs were detected: S and AH neurons. S neurons are characterized by high excitability and can exhibit rapid excitatory postsynaptic potentials, followed by a short-lived hyperpolarizing current (20–100 ms), rapidly restoring the membrane potential [66,68]. On the other hand, AH neurons exhibit large action potentials followed by a slow hyperpolarizing current (2–30 s) that makes them less excitable. NEs use more than 50 neurotransmitters in synaptic communications, from small neurotransmitters (e.g., ACh, acetylcholine, 5-HT) to neuropeptides (e.g., CGRP, calcitonin gene-related peptide, somatostatin, substance P, and VIP, vasoactive intestinal peptide) to gases (e.g., NO, nitric oxide) [67,68]. NEs are grouped into three functional classes: intrinsic sensory neurons called IPANs, muscle motor neurons, and interneurons. IPANs are large and equipped with numerous axons; they can sense mechanical, chemical, and thermal stimuli and transmit information about muscle tension state and endoluminal content to motor neurons [69], triggering reflexes that regulate motility, secretion, and blood flow. They make up about 10–30% of the neurons located in the submucosal and myenteric plexus of the small and large intestines; they are not present in the esophagus (whose motility is controlled by fibers originating from the CNS) and stomach (whose motility is under the control of vagal fibers) [69]. Motor neurons are divided into muscular and secretomotor-vasodilatory. The former (Dogiel’s type I) innervate the circular and longitudinal musculature and the muscular mucosae, determining their contraction or relaxation; they have an elongated cell body, numerous dendrites, and a single slender axon; electrophysiologically, they correspond to type S. Neurons innervating circular and longitudinal musculature have their cell bodies in the myenteric plexus and are excitatory (using ACh and TK, tachykinin, and projecting orally) or inhibitory (using NO and VIP and projecting anally) [69]. Muscle motor neurons generate, following regional stimulation, coordinated and polarized muscle responses that allow the progression of intestinal contents, i.e., induce contraction in the oral direction and relaxation in the anal direction [69]. On the other hand, secretomotor-vasodilator neurons are located mainly in the submucosal ganglia, controlling both the secretion of ions and water via ACh and the vasodilation of submucosal arterioles via VIP [66,67]. Some influence glucose transport across the mucosa of the small intestine [70], a process also regulated by vagal-like reflexes; others modulate acid secretion in the stomach [70]. Interneurons integrate sensory afferents and organize effector responses [67,68]. In the myenteric plexus, they form chains that run in ascending and descending directions. They resemble type I neurons and are S-type [68]. In the course of life, the ENS undergoes plastic changes as a spatiotemporal adaptive response to external stimuli, which arrive through sensory afferents, and to internal stimuli that come from autonomic innervation [8]. In the complex microenvironment of the gut wall lodge, different types of cells (neurons, glia, Cajal cells, muscle cells, and immune cells) can communicate with each other in synaptic or paracrine ways. This interactive plurality modulates the functional state of NEs by influencing the digestive and secretory functions of the GI tract [71]. Changes in diet and perturbations in the gut microbiome, with its metabolites and neuroactive compounds, affect the functioning of the NE and its connections with the CNS, since they alter mucosal permeability and the secretion of hormones and immune cells. In addition, NEs are vulnerable to aging-related degeneration [71].

## 3. Evidence of the Role of the Enteric Nervous System in Animal Models of Parkinson’s Disease

GI dysfunction is a common non-motor symptom of PD. While, in PD patients, it is present in 80–90% of cases and has been associated with αS aggregation and neuronal loss in the CNS, reports of GI symptoms in animal models of PD are known to vary, and the degree to which pathology in the CNS contributes to GI symptoms remains unclear [72].

PD benefits from a wide range of animal models whose diverse pharmacological, toxin, and genetic features are essential to study its etiology and neurobiology [73]. Animal models of PD rely on pharmacological or genetic approaches to simulate nigrostriatal neurodegeneration and disease pathogenesis [73]. However, much remains to be discovered and requires continuous questioning by the research community. 

**Figure 2:**
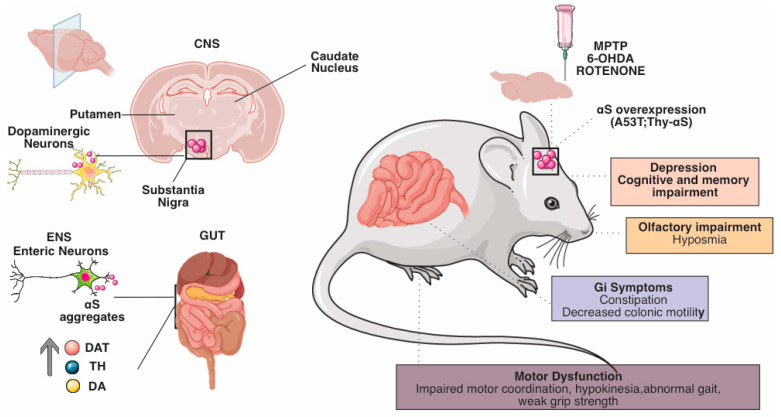
Schematic representation of the main physiological and behavioral changes in CNS and ENS of preclinical models of PD. PD is a heterogeneous disorder with varying ages of onset, symptoms, and progression rates. This heterogeneity requires the use of a variety of animal models to study different aspects of the disease. (Right) Neurotoxin-based approaches include exposure of rodents or nonhuman primates to 6-OHDA, MPTP, and agrochemicals such as the pesticide rotenone. Acute neurotoxin exposure induces motor deficits and rapid nigrostriatal dopaminergic cell death by disrupting mitochondrial function and increasing oxidative stress. Chronic neurotoxin administration induces progressive patterns that may include αS aggregates. Genetics-based approaches to modeling PD include transgenic and viral-vector-mediated models based on genes linked to monogenic PD. Among these, overexpression and introduction of preformed α-S fibrils induce toxic protein aggregates, nigrostriatal neurodegeneration, and variable motor deficits, depending on the specific model. (Left) GI dysfunction is the most common non-motor symptom of PD. Symptoms of GI dysmotility in PD include premature satiety and weight loss due to delayed gastric emptying and constipation due to altered colonic transit. We can find numerous alterations in the ENS in preclinical models of PD: neurodegeneration of NEs, which is the leading cause of behavioral and electrophysiological alterations in mouse models.

The most commonly used pharmacological models are based on neurotoxins administered to mice, rats, and nonhuman primates [74] (Figure 2).

Both neurotoxins, 1-methyl-4-phenyl-1,2,3,6-tetrahydropyridine (MPTP) and 6-hydroxydopamine (6-OHDA), consistently affect nigrostriatal dopaminergic pathways [74]. However, their impact on gut function and the CNS varies, depending on the agent, mode of administration, and assays used [75,76,77,78]. Systemic administration of MPTP in mice causes loss of dopaminergic neurons in the myenteric plexus but does not cause severe defects in GI motility [76,77]. Peripheral administration of MPTP in rats does not significantly affect the number of dopaminergic neurons and the expression of dopaminergic markers in the SNpc [79]. However, it significantly reduces tyrosine hydroxylase-immunoreactive (TH-IR) neurons in the GI tract, suggesting that the degeneration of dopaminergic neurons might start earlier than in the SNpc [48,76,79]. Parenteral administration of MPTP in mice simultaneously induces dopaminergic neurodegeneration in the ENS, which is associated with behavioral and electrophysiological alterations. Following MPTP intoxication, acceleration of motility (increased contraction) and decreased colonic relaxation are observed in response to electric field stimulation of the NE [80,81]. These complementary findings point to the altered function of enteric DA neurons. Several articles have shown that exogenous DA antagonizes colonic muscle contractility in a receptor-dependent manner [81].

Furthermore, confirming that MPTP is selectively toxic to dopaminergic neurons in the ENS, just as in the CNS, TH-positive neurons in the myenteric ganglia are reduced [82,83,84]. Most TH-positive neurons with cell bodies in the myenteric plexus can be considered dopaminergic, since adrenergic and noradrenergic inputs to the GI tract are mainly extrinsic [85]. 

Considering the neuropathological and electrophysiological findings, it is likely that dysfunction and death of dopaminergic neurons cause the transient increase in colonic motility observed after MPTP intoxication. Decreased dopaminergic inhibitory tone results in faster colonic transit due to the relative abundance of stimulatory neuronal input [80,81].

Neurotransmitters related to the GI dysfunction of PD could be involved in the intestinal dopaminergic, cholinergic, and oxidergic nitric systems [35]. To investigate the relationship between the GI dysfunction of PD and the alteration of GI neurotransmitters, 6-OHDA was microinjected into one side of the nigrostriatal system of the brain to generate an animal model of PD through the impairment of rat dopaminergic neurons, and the effect of neurotransmitter alterations in the CNS on GI function was observed [75].

GI dysfunction and changes in dopaminergic, nitric oxide synthase (NOS), and cholinergic neurons in the myenteric plexus were analyzed. Compared with control samples, 6-OHDA rats had delayed gastric emptying and constipation, which could be related to increased GI TH and decreased NOS. These symptoms were not associated with alterations in cholinergic transmitters [78].

Unfortunately, some of these studies did not analyze the submucosal plexus, making a direct comparison with more robust findings in complex PD patients [56]. Rats treated with 6-OHDA show elevated protein levels of TH and dopamine transporter (DAT) (dopaminergic markers) in both the epithelium and neurons of the GI tract, resulting in increased DA content in the gut and delayed gastric emptying [79]. In the epithelium and neurons of the GI tract, neurodegeneration of the SN by 6-OHDA increases the expression of TH and DAT proteins. It is hypothesized that the number of enteric dopaminergic neurons and cells may increase to compensate for the loss of DA in the SN in PD patients [79]. 

In contrast, the increased protein expression of TH and DAT in 6-OHDA-treated rats may increase the concentration of DA in the colon and the loss of DA in the SN, which may cause constipation [79].

Alterations in the monoaminergic system and decreased colonic motility were observed in rats microinjected with 6-OHDA in the bilateral SN [75].

DA, NE, and 5-HT play essential roles in regulating colonic motility: increased DA content, upregulation of β3-ARs, and decreased 5-HT4 receptors could contribute to the decreased spontaneous colonic contraction and constipation observed in rats with 6-OHDA [75].

Rats with lesions of SN dopaminergic neurons manifest GI dysmotility [86,87], including gastroparesis and constipation [87,88].

Animal models do not yet allow for an adequate study of how PD prodromal constipation occurs [89]. To date, there is a paucity of relevant experimental models of GI dysfunction associated with αS pathology; αS deposition in the ENS of PD patients has been reported in the myenteric and submucosal plexuses of GI tracts [90,91]. Transgenic mouse lines expressing a mutant form of human αS (A53T or A30P) under its promoter show colonic disorders similar to constipation and pathology characteristic of αS [92]. In a transgenic mouse model in which mutant human αS (A53T) was expressed under the control of the prion promoter [93], aggregates of αS were observed in the ENS prior to changes in the CNS [92]. This finding suggests that αS pathology may be initiated from the ENS and propagate to the CNS via the vagus nerve [52]. In support of this, in a transgenic mouse model, the accumulation of αS aggregates in the ENS precedes changes in the CNS [92].

Expression of human αS in the DMV, a region of the brain severely affected by PD, causes an age-related slowing in A53T mice of GI motility reminiscent of that observed in patients with PD [52,94]. The symptoms coincide with the disruption of efferent vagal processes that project from the DMV to the GI tract. This pattern parallels the pathology of postmortem specimens of PD patients and implicates the DMV as a possible mediator of GI neuropathology and symptomatology in PD [95].

However, αS mutations are only responsible for rare cases of PD [30]. Mice overexpressing wild-type human αS under the Thy-1 promoter (Thy1-αS) show increased transit time and colonic content compared with wild-type (WT) pups when tested at 12–14 months of age [96]. However, striatal dopamine loss occurs only after 14 months in Thy1-αS mice, manifesting motor and non-motor deficits, such as olfactory disturbances, as early as 2–3 months of age [97,98].

The mechanisms underlying colonic motor impairments may be related to αS overexpression in the colonic myenteric nervous system [96]. The reduced response to defecation stimuli in Thy1-αS could be related to the accumulation of αS in colonic myenteric plexuses [96].

The GI system is one of the most susceptible to environmental stressors, since it is in direct contact with environmental agents [99,100,101]. In a recent study, intra-gastric administration of rotenone in mice caused progressive αS deposition in both the ENS and CNS neurons affected by PD, such as neurons in the myenteric plexus, the vagus DMV, the spinal cord, and the sympathetic nervous system (SNS) [102]. These studies suggested that environmental stressors to the GI system could lead to αS pathology in the CNS. 

Numerous preclinical pieces of evidence associate GI symptoms in toxic models of PD based on oral administration of rotenone [99]. Previous studies have shown that orally administered rotenone exposure induces PD-like changes in the ENS and triggers PD progression throughout the nervous system to the SN [100,102]. Interestingly, the latter changes appear as early as the first moments after rotenone administration (2 months) before the onset of motor symptoms (which occur after three months of exposure in this animal model), thus mimicking the pattern of progression observed in PD patients.

In two recent studies, rotenone exposure reduced sympathetic noradrenergic [103] and vagal cholinergic gut innervation [104]. 

The mechanism by which environmental agents induce αS aggregation is unknown. However, a recent study showed that αS expression in the ENS could be upregulated by agents that cause depolarization and increase cyclic AMP levels [105].

An emerging concept in gastroenterology is that a wide range of diseases, such as motility disorders, can be partially considered enteric neuropathies. In particular, aging is associated with various motility or gut disorders, including delayed gastric emptying and longer intestinal transit time [106]. Aged rats show neuronal loss and changes in neurochemical phenotype in the ENS, which may result in motility disorders [107]. Surprisingly, along with neuronal loss, these rats exhibit dystrophic NEs that contain αS aggregates reminiscent of Lewy pathology [108].

Braak et al. hypothesized that PD originated in the gut and subsequently progressed up, as if along a ladder, along the nerves connecting the gut to the brain [91]. 

Using double transgenic mice expressing mutant αS, it is possible to observe how early alterations in ENS can be identified as early disease markers. These animal models expressing mutant αS provide an opportunity to investigate the potential role of ENS as an early marker of disease [92]. Early ENS dysfunction would not only trigger disease but facilitate the entry of deleterious factors that cause progression and spread to the CNS [12,92]. A summary of animal models exhibiting each of these characteristics is provided in Table 1.

## 4. The Possible Role of the Enteric Nervous System in Parkinson’s Disease: Clinical Evidence

**Figure 3:**
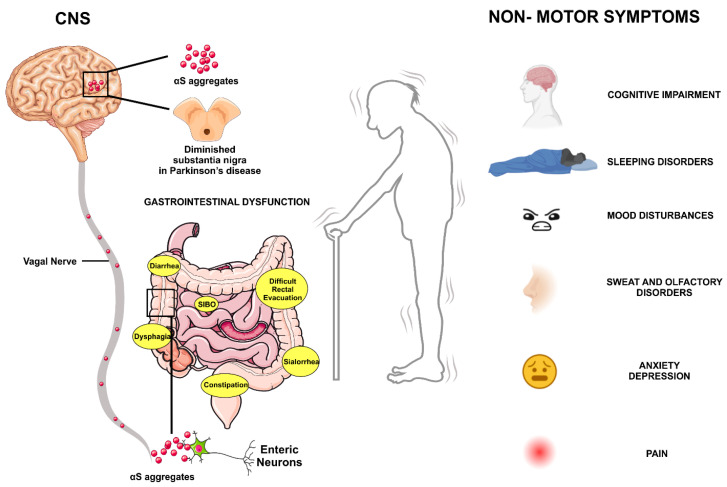
Non-motor features of Parkinson’s disease, focus on gastrointestinal symptoms. Diagnosis of PD currently depends on motor deficits, including bradykinesia, rigidity, and tremor. The motor characteristics predominantly result from the loss of dopaminergic neurons in the SNpc. However, the non-motor symptoms of PD often begin before the more visible motor symptoms. These are called “pre-motor symptoms”, such as loss of smell, depression, and constipation, which can appear years before diagnosis. The GI symptoms include excessive drooling, dysphagia, impaired gastric emptying, constipation, and impaired defecation. Moreover, alterations in the ENS levels have been reported in PD. It has been proposed that the appearance of αS aggregations beside the GI tract is an indicative tool that supports early diagnosis of PD before the onset of motor symptoms. αS has richly expressed throughout the ENS nerve plexus in healthy individuals and its growth rates with aging. Consequently, there is a need to evaluate the pathological relevance of αS carefully evaluated as a predictive biomarker of PD.

In idiopathic PD, most patients show PD-related inclusions at CNS sites and in the ENS and sympathetic ganglia where LB and Lewy neurites (LN) can be found [91]. Based on postmortem studies performed on PD patients and healthy individuals, Braak et al. proposed a pathological disease staging [109] in which PD lesions follow a specific spatiotemporal pattern, as described. These lesions start in the olfactory bulb (OB) and/or at the intestinal level, maybe in the ENS, and progress to the CNS through synaptically connected structures. This pattern seems to correspond to early non-motor symptoms in Parkinsonian patients, such as hyposmia, GI manifestations, autonomic dysfunction, and pain [110] (Figure 3).

Little is known about the ENS degenerative process in PD patients. Although PD is mainly characterized by impaired extrapyramidal motor control, clinical studies have revealed delayed gastric emptying, external anal sphincter dystonia causing difficult rectal evacuation, and general slow-transit constipation caused by local loss of dopaminergic neurons [111,112,113]. Many researchers have recently studied the expression and modifications of enteric αS in PD patients, with controversial results. Although some have found increased inclusions of αS and phosphorylated αS in the above areas compared with control subjects [50], others point attention to high variability among patients [99]. 

All these hypotheses take advantage of clinical observation of both the prodromal symptoms and the non-motor ones [114,115,116], assuming that the involvement of the dopaminergic system in PD neurodegenerative process starts at the level of the DMV with a pattern of periphery–center (bottom–top) [115].

This process would initially occur in the enteric system (signs: constipation slow and transit alteration) and then progress to the brainstem with hypo/anosmia and sleep disturbances up to the mesencephalon (SNpc) with the appearance of the cardinal symptoms (rigidity, bradykinesia, tremor, and postural instability); finally, it would involve the cerebral cortex with the appearance of cognitive and behavioral disturbances [117]. Braak predicted six stages of the disease, of which only the third one involves the CNS with the appearance of motor symptoms [52,109], in a sort of dominos game [118]. An exciting and innovative hypothesis has shifted the interest of researchers in the last 20 years toward the discovery of early biomarkers [119]. The big challenge was to identify a potential pathogen capable of passing the mucosal barrier of the GI tract and, via postganglionic ENs, entering the CNS along unmyelinated preganglionic fibers generated from the viscero-motor projection cells of the vagus nerve [120]. There is also epidemiological evidence that complete but not partial vagotomy may protect against later PD [121,122].

Other studies suggested that not all cases of PD start in the ENS. Autopsy studies have shown that a minority of cases showing Lewy pathology do not present pathological inclusions in the DMV. Moreover, some cases display a distribution of αS inclusions that can be limbic-predominant, revealing less pathology in the brainstem [123,124,125]. Therefore, different subtypes of PD have been proposed according to these criteria: (i) a body-first (bottom-up) subtype, in which the disease starts in the enteric or peripheral autonomic nervous system and arises, via the sympathetic connectome of the vagus nerve, to the CNS [126]. This phenotype shows prolonged intestinal transit and constipation as prodromic symptoms; (ii) a brain-first (top-down) subtype in which the αS pathology originates in the brain or via the OB and descends to the peripheral autonomic nervous system. This phenotype shows hyposmia and sleep disturbances as prodromic signs [127]. These differences reflect the high variability between patients’ phenotype and clinical signs in a “puzzle game” that PD seems to be.

The GBA comprises different functional, neuroendocrine, and neuroimmune systems, including the hypothalamic–pituitary–adrenal axis, the ENS through the sympathetic and parasympathetic systems, the vagus nerve, intestinal immune cells, and intestinal microbiota [128].

Approximately 100 trillion microbes in the human gut are involved in food fermentation, metabolic, and immune maturity. Many play a central role in developing the ENS and CNS and in the modulation of the pathogenesis of metabolic, neurodegenerative, and neurodevelopmental disorders [129].

The gut-hosted bacteria can impact brain function via different pathways. These bottom-up pathways include direct absorption through the gut–blood/lymphatic–brain pathways, as well as local signaling in the gut to prime immune cells, and the vagal retrograde transport pathways [130]. It was demonstrated that microbe-secreted products such as neurotransmitters, including catecholamines, GABA, 5-HT, and gut metabolites transit through the gut–blood and blood–brain barriers and elicit an immune response that changes the profiles of plasma proteomics and brain neurochemistry. In a different network, bacterial metabolites can activate immune cells [131,132]. A third pathway represented by the vagal route was identified too. Evidence shows that the vagus nerve can transport αS from the gut to the brain [133].

Studies performed by amplifying the rRNA gene or by “metagenomic sequencing” revealed changes in the intestinal microbiota of PD patients compared to healthy controls [134]. Modifications in the intestinal microbiome also correlate with PD progression. For example, a decrease in the microbiota producing short-chain fatty acids and an increase in proinflammatory bacteria seem to correlate with motor and cognitive severity in PD patients [135]. A longitudinal follow-up clinical study showed that a decreased amount of Roseburia intestinal bacteria is linked to a rapid progression of both motor and non-motor symptoms of PD. Moreover, a reduced amount of short-chain fatty-acid-producing bacteria, such as Fusicatenibacter and Faecalibacterium, is correlated to an increase in fecal inflammatory calprotectin levels in Parkinsonian patients [136].

Systemic and fecal inflammatory markers IFN-γ, TNF-α, and neutrophil gelatinase-associated lipocalin were also associated with an elevated expression of Bacteroides and Bifidobacterium in PD patients [41]. Thus, the intestinal microbiota composition in PD patients appears to influence pharmacological treatment responses [135]. A growing body of evidence supports the role of the microbiome in the pharmacokinetics of drugs used in PD treatment; at the same time, the drugs can alter the gut microbiome’s composition [137]. This evidence highlights the concept that the intestinal microbiome may influence the treatment efficacy and the development of potential modified response to levodopa therapy [135]. 

In conclusion, in the very early stages of PD before CNS pathology, accumulation of enteric αS [138] may promote activation of immune/inflammatory signaling, including canonical caspase-1-dependent inflammasome pathways [139], resulting in a massive release of IL-1β, which, in turn, alters the intestinal epithelial barrier through the activation of IL-1 receptors on intestinal epithelial cells [139]. In this context, intestinal inflammation and altered intestinal epithelial barrier can induce changes in short-chain fatty acid levels, characterized by alterations in butyrate levels, which could contribute to the impairment of the intestinal epithelial barrier [140]. They can also cause an increase in the concentration of circulating lipopolysaccharide, contributing further to activating the intestinal immune/inflammatory pathways. This would induce a vicious circle that could bring the chronicization of inflammatory processes with the appearance of intestinal symptoms and brain pathology [141] (Table 2).

## 5. New Therapeutic Approach Targeting the Enteric Nervous System

The lessening of dopaminergic striatal and nigral innervation alters local microcircuits [142,143]. The emerging scenarios concerning enteric involvement in PD pathogenesis offer a new therapeutic approach.

The nonpharmacological approach based on the increase in enteric system motility has been well defined in the last few years. For example, a high fiber diet, appropriate fluid intake, and psyllium can represent an excellent approach to counteract the slowing of bowel pain in many PD patients, as well as exercise and physical activity directed to stimulate autonomic symptoms (impaired gastric motility, dysphagia, constipation, and bowel incontinence) [34]. These approaches are based on evidence that exercise may also change dopamine receptor availability in animal models of PD and patients [42,144].

Adjustment of anticholinergics and dopaminergic agents used for PD therapy can contribute to relieving intestinal and motor symptoms by demonstrating the connection between enteric and CNS [40]. Many other approaches were proposed to treat the comorbidity of PD, considering that gut dysfunction may contribute to the symptomatic fluctuation in PD patients. 

The microbiome is also well discussed in many other papers focusing on the enteric flora to explain different phenomena. Many different types of microbiomes have been found in different PD patients, which does not allow a unique key for reading, confirming the complexity of PD and the possibility that an inadequate therapeutic approach is used.

## 6. Discussion

PD is a frequent neurodegenerative disorder characterized by a constellation of clinical manifestations: apart from classic motor symptoms, patients also often experience non-motor manifestations, including hyposmia, sleep disturbances, depression, dementia, and GI dysfunction [144,145,146,147,148], some of them could appear even decades before the onset of motor signs [149,150,151,152,153]. 

GI dysfunction often occurs in the early stages of the disease [154]. This observation and the detection of misfolded αS protein in the ENS of PD patients [145,146] have directed interest toward the hypothesis that the disorder may originate in the gut. Indeed, it has become increasingly evident that, in PD, the neurodegenerative process involves several structures even distant from the CNS, such as the ENS, which is the dense neural network of neurons and glial cells regulating and co-ordinating gut function and motility, referred to as the “brain in the gut” or “second brain” [32,34,35,36,148]. Following this hypothesis, several studies have investigated the role of ENS in PD [91]. 

On the one hand, Lewy pathology could be induced in the ENS and transported to the CNS via the vagal nerve. The aggregation and propagation of enteric-derived αS probably indicate an early pathological stage that could subsequently initiate the motor and non-motor symptoms characteristic of PD. 

On the other hand, the gut microbiota might also play a role through the effect of different molecules and proteins produced by gut bacteria that can act locally or be transported to the CNS through the vagus nerve fibers. This transport of substances between the gut and CNS has been verified in pathological conditions [5,20], finding implications in PD. Dysbiosis of the microbiota leads to an imbalance between beneficial and harmful microbial metabolites, causing increased intestinal permeability and inflammation and systemic inflammation. αS aggregates in the intestine likely induce enteric pathology and dysfunction, which can trigger enteric inflammation, dysbiosis, and intestinal hyperpermeability. The triggered inflammatory state impacts the CNS and promotes PD pathology. 

In this review, we aim to discuss the ENS involvement in the pathophysiology of PD by providing evidence from preclinical and clinical studies. 

Until April 2023, there have been 66,523 articles about the etiology of PD on the Pub-Med database. Of these papers, 13,060 are reviews. However, a substantial limitation is that only 0.99% of articles support the gut–brain axis theory, with only 546 works discussing the ENS and PD and 1119 articles focusing on PD and the microbiome. Current research focuses mainly on the microbiome rather than the relationships between the autonomic nervous system and the CNS, which probably underlie all etiological processes. 

Therefore, our review attempted to go beyond the role of the microbiota, focusing mainly on other possible players in PD pathology, such as the ENS. Indeed, we believe that the field of microbiome research is complicated and highly heterogeneous. Moreover, the multitude of factors mediating the potential influence of the gut microbiome on PD is influenced by everyone’s dietary and lifestyle habits, levels of inflammation, comorbidities, and use of supplements or medications [136]. Different compositions of the gut microbiomes in PD patients represent a limiting factor in interpreting results. In addition, ethnological, cultural, and lifestyle differences may cause heterogeneous results among studies, posing a limitation for further research. We believe more studies from different nations and regions are needed to explore the relationship between PD and GI diseases. 

Although alterations in the GI tract have been highlighted in the pathogenesis of PD, the exact mechanism linking enteric inflammation and neurodegeneration remains to be elucidated. This would significantly aid in early diagnosis and intervention to slow or halt disease progression. Indeed, there is currently no therapy available to cure PD, and the early stages of the disease are probably best suited for personalized disease-modifying interventions. In this regard, animal models may represent an essential tool to study the pathogenesis of PD, as they offer the possibility of simultaneously observing behavioral abnormalities, in vivo imaging, and pathological assessments. However, as with clinical trials, preclinical research still has limitations, since animal models of PD do not accurately recapitulate human PD [155]. Moreover, animal models that recapitulate the prodromal stages of the disease still need to be developed [156] and few approaches are available to study alterations of the gut microbiome, inflammatory processes, and disease progression [157]. The limited availability of animal models that can recapitulate prodromal PD and reproduce both peripheral and central pathology has dramatically slowed the understanding of PD pathogenesis, including our comprehension of the precocious involvement of the autonomic nervous system, as well as of other non-motor symptoms that precede motor signs by several years. Nonetheless, different PD animal models may offer important insights into the role of the ENS and the gut microbiome. 

Many approaches have been proposed to treat the comorbidity of PD, considering that bowel dysfunction may contribute to symptomatic fluctuation in PD patients. Based on this evidence, the ENS may be an excellent target to investigate some multifactorial aspects of PD and a potential biomarker for early diagnosis of PD. Identifying a reliable preclinical PD biomarker would be critical, enabling early intervention that could slow or prevent the disease [149,150]. A better understanding of the ENS and its relationships beyond the gut microbiome could represent a new scenario to better characterize a disease of which only the final stages are known but which probably represents the sum of numerous insults occurring over a lifetime of more than 20 years.

## 7. Conclusions

PD presents a significant challenge, unraveling with misrecognized symptoms that appear decades before motor features. PD studies have focused primarily on the CNS and associated motor dysfunction; however, the peripheral nervous system, including the ENS, is gaining prominence in the field of PD. Despite technological advances in neuroimaging, no fully validated biomarker is available for PD. There is an urgent need to identify biomarkers to differentiate PD from related disorders and assess disease severity and progression. From a clinical perspective, NEs and enteric glia could represent important new targets for the pharmacological treatment of neurodegenerative diseases.

Future studies should focus on this possibility, especially given the relative ease of studying these cells in humans. Considering the clinical and experimental evidence, the authors propose that Parkinsonism is only one aspect of a complex and multifaceted disorder, representing the last phase of a neuropathological process that begins at a young age in ENS.

## Figures and Tables

**Table 1:** Pathological features identified in animal models of PD. The table summarizes the significant alterations found in murine models of PD. The legend of the abbreviations is listed below.

PD Model	Affected Neuron Types	GI Symptoms	Alteration Biomarker	References
**MPTP mice**	Loss of dopaminergic neurons in the myenteric plexus.	Absence of severe defects in GI motility. Increased contraction and decreased relaxation of colon muscle in response to electric field stimulation of NEs.	Nd	[76,77]
**MPTP rats** (Peripheral administration)	Unaltered number of dopaminergic neurons in the SNpc. Presence of TH-IR neurons in the GI tract.	Nd	Unaltered expression of dopaminergic markers in the SNpc.	[79] [48,76,79]
**6-OHDA rats**	Alterations in the monoaminergic and cholinergic system.	Delayed gastric emptying and constipation, which could be related to increased GI TH and decreased NOS. Increased DA concentration in the colon, which is more likely to cause constipation. Decreased colonic motility.	Unaltered cholinergic transmitters. Elevated protein levels of TH and DAT both in the epithelium and neurons of the GI tract, resulting in increased DA content in the gut and delayed gastric emptying.	[75] [78,79] [86,87,88]
**A53T mice** (Expressing a mutant form of human αS)	Disruption of efferent vagal processes that project from the DMV to the GI tract.	Related slowing of GI motility caused by expression of human αS in the DMV.	Accumulation of αS aggregates in the ENS before changes in the CNS.	[92] [52,94] [95]
**Thy1-**α**S mice**	Nd	Striatal dopamine loss only after 14 months: manifesting motor and non-motor deficits, such as olfactory disturbances, as early as 2–3 months of age.	Increased transit time and colonic content. Overexpression of αS in the colonic myenteric nervous system. Reduced response to defecation stimuli.	[96] [97,98]
**Rotenone** **mice model**	Reduced sympathetic noradrenergic and vagal cholinergic gut innervation.	Aggregates of αS in both ENS neurons of the myenteric plexus and at the level of the DMV, spinal cord, and SNS.	Nd	[99] [102] [103] [104]
**Fischer 344 rat**	Neuronal loss and changes in neurochemical phenotype in the ENS.	Dystrophic enteric neurons that contain αS aggregates reminiscent of Lewy pathology.	Motility disorders	[108]

Parkinson’s disease (PD); enteric neurons (NEs); 1-methyl-4-phenyl-1,2,3,6-tetrahydropyridine (MPTP); substantia nigra pars compacta (SNpc), tyrosine hydroxylase-immunoreactive (TH-IR); gastrointestinal tract (GI), tyrosine hydroxylase (TH); enteric nervous system (ENS); central nervous system (CNS); sympathetic nervous system (SNS); alpha-synuclein (αS); 6-hydroxydopamine (6-OHDA); dorsal motor nucleus of the vagus nerve (DMV); dopamine transporter (DAT); dopamine (DA); nitric oxide synthase (NOS); Not declared (Nd).

**Table 2:** Pathological features identified in PD patients.

PD Symptoms	Affected Neuron Types	GI Symptoms	Alteration Biomarker	References
Nd	Nd	Gastric emptying. Difficult rectal evacuation. Slow transit constipation.	Nd	[111,112,113]
Hypo/anosmia. Sleep disturbances. Rigidity, bradykinesia, tremor, and postural instability. Cognitive and behavioral disturbances.	Neurodegenerative process starting in the DMV with a pattern of periphery–center (bottom–top).	Nd	Increased inclusions of αS and phosphorylated αS.	[115] [117]
Nd	Nd	Prolonged intestinal transit and constipation.	Minority of cases with Lewy pathology without pathological inclusions in the DMV. Limbic-predominant distribution of αS inclusions with less pathology in the brainstem.	[123,124] [126]
Motor and cognitive symptoms.	Nd	Nd	Decrease in the short-chain fatty acids, including Fusicatenibacter and Faecalibacterium. Increase in proinflammatory bacteria.	[135] [136]
Nd	Nd	Nd	Systemic and fecal inflammatory markers IFN-γ, TNF-α, and neutrophil gelatinase-associated lipocalin, associated with an elevated expression of Bacteroides and Bifidobacterium.	[41]
Nd	Nd	Alteration in intestinal epithelial barrier.	Accumulation of enteric αS. Activation of immune/inflammatory signaling, including canonical caspase-1- dependent inflammasome pathways. Massive release of IL-1β.	[138] [139] [140]

Alpha-synuclein (αS); dorsal motor nucleus of the vagus nerve (DMV); tumor necrosis factor-alpha (TNF-α); interferon-gamma (IFN-γ); interleukin-1β (IL-1β); Not declared (Nd).

## Data Availability

All the data shown in this paper are available in PubMed Library. The authors created all representative draws appositely and are available on request.

## Abbreviations

(ach)	Acetylcholine
αS	α-synuclein
CNS	Central nervous system
DA	Dopamine
DAT	Dopamine transporter
DMV	Dorsal motor nucleus of the vagus nerve
ENS	Enteric nervous system
NEs	Enteric neurons
GI	Gastrointestinal tract
GBA	Gut–brain axis
GABA	G-aminobutyric acid
IFN-γ	Interferon-gamma
IL-1β	Interleukin-1β
Thy1-αS	Human αs under the thy-1 promoter
6-OHDA	6-hydroxydopamine
LB	Lewy bodies
MPTP	1-methyl-4-phenyl-1,2,3,6-tetrahydropyridine
NO	Nitric oxide
NOS	Nitric oxide synthase
OB	Olfactory bulb
PD	Parkinson’s disease
SNS	Sympathetic Nervous System
SN	Substantia nigra
SNpc	Substantia nigra pars compacta
5-HT	5-hydroxytryptamine or serotonine
TNF-α	Tumor necrosis factor-alpha
TH	Tyrosine hydroxylase
TH-IR	Tyrosine Hydroxylase-Immunoreactive
VIP	Vasoactive intestinal peptide
WT	Wild-type

## References

[1] Endres K., Schäfer K.-H. (2018). Influence of commensal microbiota on the enteric nervous system and its role in neurodegenerative diseases. J. Innate Immun..

[2] Clemente J.C., Ursell L.K., Parfrey L.W., Knight R. (2012). The impact of the gut microbiota on human health: An integrative view. Cell.

[3] Bercik P., Denou E., Collins J., Jackson W., Lu J., Jury J., Deng Y., Blennerhassett P., Macri J., McCoy K.D. (2011). The intestinal microbiota affect central levels of brain-derived neurotropic factor and behavior in mice. Gastroenterology.

[4] Sampson T.R., Mazmanian S.K. (2015). Control of brain development, function, and behavior by the microbiome. Cell Host Microbe.

[5] Carabotti M., Scirocco A., Maselli M.A., Severi C. (2015). The gut-brain axis: Interactions between enteric microbiota, central and enteric nervous systems. Ann. Gastroenterol..

[6] Kabouridis P.S., Lasrado R., McCallum S., Chng S.H., Snippert H.J., Clevers H., Pettersson S., Pachnis V. (2015). Microbiota controls the homeostasis of glial cells in the gut lamina propria. Neuron.

[7] Belkaid Y., Hand T.W. (2014). Role of the microbiota in immunity and inflammation. Cell.

[8] Furness J.B. (2008). The Enteric Nervous System.

[9] Sasselli V., Pachnis V., Burns A.J. (2012). The enteric nervous system. Dev. Biol..

[10] Furness J.B. (2012). The enteric nervous system and neurogastroenterology. Nat. Rev. Gastroenterol. Hepatol..

[11] Gershon M.D. (1999). The enteric nervous system: A second brain. Hosp. Pract..

[12] Natale G., Pasquali L., Paparelli A., Fornai F. (2011). Parallel manifestations of neuropathologies in the enteric and central nervous systems. Neurogastroenterol. Motil..

[13] Furness J.B. (2006). The organisation of the autonomic nervous system: Peripheral connections. Auton. Neurosci..

[14] Cryan J.F., O’Riordan K.J., Cowan C.S.M., Sandhu K.V., Bastiaanssen T.F.S., Boehme M., Codagnone M.G., Cussotto S., Fulling C., Golubeva A.V. (2019). The Microbiota-Gut-Brain Axis. Physiol. Rev..

[15] Wang H.X., Wang Y.P. (2016). Gut Microbiota-brain Axis. Chin. Med. J..

[16] Jaggar M., Rea K., Spichak S., Dinan T.G., Cryan J.F. (2020). You’ve got male: Sex and the microbiota-gut-brain axis across the lifespan. Front. Neuroendocrinol..

[17] Bauer P.V., Hamr S.C., Duca F.A. (2016). Regulation of energy balance by a gut-brain axis and involvement of the gut microbiota. Cell. Mol. Life Sci..

[18] Margolis K.G., Cryan J.F., Mayer E.A. (2021). The Microbiota-Gut-Brain Axis: From Motility to Mood. Gastroenterology.

[19] Rhee S.H., Pothoulakis C., Mayer E.A. (2009). Principles and clinical implications of the brain-gut-enteric microbiota axis. Nat. Rev. Gastroenterol. Hepatol..

[20] Kasarello K., Cudnoch-Jedrzejewska A., Czarzasta K. (2023). Communication of gut microbiota and brain via immune and neuroendocrine signaling. Front. Microbiol..

[21] Mayer E.A., Savidge T., Shulman R.J. (2014). Brain-gut microbiome interactions and functional bowel disorders. Gastroenterology.

[22] Varesi A., Pierella E., Romeo M., Piccini G.B., Alfano C., Bjørklund G., Oppong A., Ricevuti G., Esposito C., Chirumbolo S. (2022). The potential role of gut microbiota in alzheimer’s disease: From diagnosis to treatment. Nutrients.

[23] Quigley E.M.M. (2017). Microbiota-Brain-Gut Axis and Neurodegenerative Diseases. Curr. Neurol. Neurosci. Rep..

[24] Sidransky E., Lopez G. (2012). The link between the GBA gene and parkinsonism. Lancet Neurol..

[25] Schirinzi T., Martella G., Pisani A. (2016). Double hit mouse model of Parkinson’s disease. Oncotarget.

[26] Martella G., Madeo G., Maltese M., Vanni V., Puglisi F., Ferraro E., Schirinzi T., Valente E.M., Bonanni L., Shen J. (2016). Exposure to low-dose rotenone precipitates synaptic plasticity alterations in PINK1 heterozygous knockout mice. Neurobiol. Dis..

[27] Dickson D.W. (2012). Parkinson’s disease and parkinsonism: Neuropathology. Cold Spring Harb. Perspect. Med..

[28] Hawkes C.H., Del Tredici K., Braak H. (2007). Parkinson’s disease: A dual-hit hypothesis. Neuropathol. Appl. Neurobiol..

[29] Tanner C.M., Goldman S.M. (1996). Epidemiology of Parkinson’s disease. Neurol. Clin..

[30] Vance J.M., Ali S., Bradley W.G., Singer C., Di Monte D.A. (2010). Gene-environment interactions in Parkinson’s disease and other forms of parkinsonism. Neurotoxicology.

[31] Kline E.M., Houser M.C., Herrick M.K., Seibler P., Klein C., West A., Tansey M.G. (2021). Genetic and environmental factors in parkinson’s disease converge on immune function and inflammation. Mov. Disord..

[32] Poewe W. (2008). Non-motor symptoms in Parkinson’s disease. Eur. J. Neurol..

[33] Noyce A.J., Bestwick J.P., Silveira-Moriyama L., Hawkes C.H., Giovannoni G., Lees A.J., Schrag A. (2012). Meta-analysis of early nonmotor features and risk factors for Parkinson disease. Ann. Neurol..

[34] Amara A.W., Memon A.A. (2018). Effects of Exercise on Non-motor Symptoms in Parkinson’s Disease. Clin. Ther..

[35] Postuma R.B., Aarsland D., Barone P., Burn D.J., Hawkes C.H., Oertel W., Ziemssen T. (2012). Identifying prodromal Parkinson’s disease: Pre-motor disorders in Parkinson’s disease. Mov. Disord..

[36] Martinez-Martin P., Rodriguez-Blazquez C., Kurtis M.M., Chaudhuri K.R. (2011). NMSS Validation Group The impact of non-motor symptoms on health-related quality of life of patients with Parkinson’s disease. Mov. Disord..

[37] Battaglia S., Nazzi C., Thayer J.F. (2023). Fear-induced bradycardia in mental disorders: Foundations, current advances, future perspectives. Neurosci. Biobehav. Rev..

[38] Battaglia S., Di Fazio C., Vicario C.M., Avenanti A. (2023). Neuropharmacological Modulation of N-methyl-D-aspartate, Noradrenaline and Endocannabinoid Receptors in Fear Extinction Learning: Synaptic Transmission and Plasticity. Int. J. Mol. Sci..

[39] Tan A.H., Lim S.Y., Lang A.E. (2022). The microbiome-gut-brain axis in Parkinson disease—From basic research to the clinic. Nat. Rev. Neurol..

[40] Mukherjee A., Biswas A., Das S.K. (2016). Gut dysfunction in Parkinson’s disease. World J. Gastroenterol..

[41] Zeng J., Wang X., Pan F., Mao Z. (2022). The relationship between Parkinson’s disease and gastrointestinal diseases. Front. Aging Neurosci..

[42] Bhidayasiri R., Phuenpathom W., Tan A.H., Leta V., Phumphid S., Chaudhuri K.R., Pal P.K. (2022). Management of dysphagia and gastroparesis in Parkinson’s disease in real-world clinical practice—Balancing pharmacological and non-pharmacological approaches. Front. Aging Neurosci..

[43] Chen Z., Li G., Liu J. (2020). Autonomic dysfunction in Parkinson’s disease: Implications for pathophysiology, diagnosis, and treatment. Neurobiol. Dis..

[44] Chiang H.-L., Lin C.-H. (2019). Altered gut microbiome and intestinal pathology in parkinson’s disease. J. Mov. Disord..

[45] Devos D., Lebouvier T., Lardeux B., Biraud M., Rouaud T., Pouclet H., Coron E., Bruley des Varannes S., Naveilhan P., Nguyen J.-M. (2013). Colonic inflammation in Parkinson’s disease. Neurobiol. Dis..

[46] Cersosimo M.G., Benarroch E.E. (2012). Pathological correlates of gastrointestinal dysfunction in Parkinson’s disease. Neurobiol. Dis..

[47] Stocchi F., Torti M. (2017). Constipation in parkinson’s disease. Int. Rev. Neurobiol..

[48] Singaram C., Ashraf W., Gaumnitz E.A., Torbey C., Sengupta A., Pfeiffer R., Quigley E.M. (1995). Dopaminergic defect of enteric nervous system in Parkinson’s disease patients with chronic constipation. Lancet.

[49] Pfeiffer R.F., Isaacson S.H., Pahwa R. (2020). Clinical implications of gastric complications on levodopa treatment in Parkinson’s disease. Park. Relat. Disord..

[50] Lebouvier T., Neunlist M., Bruley des Varannes S., Coron E., Drouard A., N’Guyen J.-M., Chaumette T., Tasselli M., Paillusson S., Flamand M. (2010). Colonic biopsies to assess the neuropathology of Parkinson’s disease and its relationship with symptoms. PLoS ONE.

[51] Zheng H., Shi C., Luo H., Fan L., Yang Z., Hu X., Zhang Z., Zhang S., Hu Z., Fan Y. (2021). α-Synuclein in Parkinson’s Disease: Does a Prion-like Mechanism of Propagation from Periphery to the Brain Play a Role?. Neuroscientist.

[52] Braak H., Del Tredici K., Rüb U., de Vos R.A.I., Jansen Steur E.N.H., Braak E. (2003). Staging of brain pathology related to sporadic Parkinson’s disease. Neurobiol. Aging.

[53] Arotcarena M.-L., Dovero S., Prigent A., Bourdenx M., Camus S., Porras G., Thiolat M.-L., Tasselli M., Aubert P., Kruse N. (2020). Bidirectional gut-to-brain and brain-to-gut propagation of synucleinopathy in non-human primates. Brain.

[54] Natale G., Pasquali L., Ruggieri S., Paparelli A., Fornai F. (2008). Parkinson’s disease and the gut: A well known clinical association in need of an effective cure and explanation. Neurogastroenterol. Motil..

[55] Leclair-Visonneau L., Neunlist M., Derkinderen P., Lebouvier T. (2020). The gut in Parkinson’s disease: Bottom-up, top-down, or neither?. Neurogastroenterol. Motil..

[56] Chalazonitis A., Rao M. (2018). Enteric nervous system manifestations of neurodegenerative disease. Brain Res..

[57] Menozzi E., Macnaughtan J., Schapira A.H.V. (2021). The gut-brain axis and Parkinson disease: Clinical and pathogenetic relevance. Ann. Med..

[58] Berg D., Borghammer P., Fereshtehnejad S.-M., Heinzel S., Horsager J., Schaeffer E., Postuma R.B. (2021). Prodromal Parkinson disease subtypes—Key to understanding heterogeneity. Nat. Rev. Neurol..

[59] Elfil M., Kamel S., Kandil M., Koo B.B., Schaefer S.M. (2020). Implications of the gut microbiome in parkinson’s disease. Mov. Disord..

[60] Klann E.M., Dissanayake U., Gurrala A., Farrer M., Shukla A.W., Ramirez-Zamora A., Mai V., Vedam-Mai V. (2021). The Gut-Brain Axis and Its Relation to Parkinson’s Disease: A Review. Front. Aging Neurosci..

[61] Ma Z.S. (2020). Heterogeneity-disease relationship in the human microbiome-associated diseases. FEMS Microbiol. Ecol..

[62] Natale G., Ryskalin L., Morucci G., Lazzeri G., Frati A., Fornai F. (2021). The baseline structure of the enteric nervous system and its role in parkinson’s disease. Life.

[63] Brodal P. (2004). The Central Nervous System: Structure and Function.

[64] Cussotto S., Strain C.R., Fouhy F., Strain R.G., Peterson V.L., Clarke G., Stanton C., Dinan T.G., Cryan J.F. (2019). Differential effects of psychotropic drugs on microbiome composition and gastrointestinal function. Psychopharmacology.

[65] The Enteric Nervous System and Regulation of Intestinal Motility—ProQuest. https://www.proquest.com/docview/222539969?pq-origsite=gscholar&fromopenview=true.

[66] Brehmer A. (2006). Structure of Enteric Neurons.

[67] Costa M., Furness J.B., Gibbins I.L. (1986). Chapter 15 Chemical coding of enteric neurons. Progress in Brain Research.

[68] Furness J.B., Costa M. (1980). Types of nerves in the enteric nervous system. Commentaries in the Neurosciences.

[69] Furness J.B., Callaghan B.P., Rivera L.R., Cho H.-J. (2014). The enteric nervous system and gastrointestinal innervation: Integrated local and central control. Adv. Exp. Med. Biol..

[70] Shirazi-Beechey S.P., Moran A.W., Batchelor D.J., Daly K., Al-Rammahi M. (2011). Glucose sensing and signalling; regulation of intestinal glucose transport. Proc. Nutr. Soc..

[71] Saffrey M.J. (2013). Cellular changes in the enteric nervous system during ageing. Dev. Biol..

[72] McQuade R.M., Singleton L.M., Wu H., Lee S., Constable R., Di Natale M., Ringuet M.T., Berger J.P., Kauhausen J., Parish C.L. (2021). The association of enteric neuropathy with gut phenotypes in acute and progressive models of Parkinson’s disease. Sci. Rep..

[73] Lama J., Buhidma Y., Fletcher E.J.R., Duty S. (2021). Animal models of Parkinson’s disease: A guide to selecting the optimal model for your research. Neuronal Signal..

[74] Tieu K. (2011). A guide to neurotoxic animal models of Parkinson’s disease. Cold Spring Harb. Perspect. Med..

[75] Zhang X., Li Y., Liu C., Fan R., Wang P., Zheng L., Hong F., Feng X., Zhang Y., Li L. (2015). Alteration of enteric monoamines with monoamine receptors and colonic dysmotility in 6-hydroxydopamine-induced Parkinson’s disease rats. Transl. Res..

[76] Anderson G., Noorian A.R., Taylor G., Anitha M., Bernhard D., Srinivasan S., Greene J.G. (2007). Loss of enteric dopaminergic neurons and associated changes in colon motility in an MPTP mouse model of Parkinson’s disease. Exp. Neurol..

[77] Chaumette T., Lebouvier T., Aubert P., Lardeux B., Qin C., Li Q., Accary D., Bézard E., Bruley des Varannes S., Derkinderen P. (2009). Neurochemical plasticity in the enteric nervous system of a primate animal model of experimental Parkinsonism. Neurogastroenterol. Motil..

[78] Zhu H.C., Zhao J., Luo C.Y., Li Q.Q. (2012). Gastrointestinal dysfunction in a Parkinson’s disease rat model and the changes of dopaminergic, nitric oxidergic, and cholinergic neurotransmitters in myenteric plexus. J. Mol. Neurosci..

[79] Tian Y.M., Chen X., Luo D.Z., Zhang X.H., Xue H., Zheng L.F., Yang N., Wang X.M., Zhu J.X. (2008). Alteration of dopaminergic markers in gastrointestinal tract of different rodent models of Parkinson’s disease. Neuroscience.

[80] Li Z.S., Schmauss C., Cuenca A., Ratcliffe E., Gershon M.D. (2006). Physiological modulation of intestinal motility by enteric dopaminergic neurons and the D2 receptor: Analysis of dopamine receptor expression, location, development, and function in wild-type and knock-out mice. J. Neurosci..

[81] Walker J.K., Gainetdinov R.R., Mangel A.W., Caron M.G., Shetzline M.A. (2000). Mice lacking the dopamine transporter display altered regulation of distal colonic motility. Am. J. Physiol. Gastrointest. Liver Physiol..

[82] Bové J., Prou D., Perier C., Przedborski S. (2005). Toxin-induced models of Parkinson’s disease. NeuroRx.

[83] Jackson-Lewis V., Jakowec M., Burke R.E., Przedborski S. (1995). Time course and morphology of dopaminergic neuronal death caused by the neurotoxin 1-methyl-4-phenyl-1,2,3,6-tetrahydropyridine. Neurodegeneration.

[84] Heikkila R.E., Hess A., Duvoisin R.C. (1984). Dopaminergic neurotoxicity of 1-methyl-4-phenyl-1,2,5,6-tetrahydropyridine in mice. Science.

[85] Li Z.S., Pham T.D., Tamir H., Chen J.J., Gershon M.D. (2004). Enteric dopaminergic neurons: Definition, developmental lineage, and effects of extrinsic denervation. J. Neurosci..

[86] Wakabayashi K., Takahashi H., Ohama E., Ikuta F. (1990). Parkinson’s disease: An immunohistochemical study of Lewy body-containing neurons in the enteric nervous system. Acta Neuropathol..

[87] Colucci M., Cervio M., Faniglione M., De Angelis S., Pajoro M., Levandis G., Tassorelli C., Blandini F., Feletti F., De Giorgio R. (2012). Intestinal dysmotility and enteric neurochemical changes in a Parkinson’s disease rat model. Auton. Neurosci..

[88] Zheng L.F., Song J., Fan R.F., Chen C.L., Ren Q.Z., Zhang X.L., Feng X.Y., Zhang Y., Li L.S., De Giorgio R. (2014). The role of the vagal pathway and gastric dopamine in the gastroparesis of rats after a 6-hydroxydopamine microinjection in the substantia nigra. Acta Physiol..

[89] Rota L., Pellegrini C., Benvenuti L., Antonioli L., Fornai M., Blandizzi C., Cattaneo A., Colla E. (2019). Constipation, deficit in colon contractions and alpha-synuclein inclusions within the colon precede motor abnormalities and neurodegeneration in the central nervous system in a mouse model of alpha-synucleinopathy. Transl. Neurodegener..

[90] Qualman S.J., Haupt H.M., Yang P., Hamilton S.R. (1984). Esophageal Lewy bodies associated with ganglion cell loss in achalasia. Gastroenterology.

[91] Braak H., de Vos R.A.I., Bohl J., Del Tredici K. (2006). Gastric alpha-synuclein immunoreactive inclusions in Meissner’s and Auerbach’s plexuses in cases staged for Parkinson’s disease-related brain pathology. Neurosci. Lett..

[92] Kuo Y.-M., Li Z., Jiao Y., Gaborit N., Pani A.K., Orrison B.M., Bruneau B.G., Giasson B.I., Smeyne R.J., Gershon M.D. (2010). Extensive enteric nervous system abnormalities in mice transgenic for artificial chromosomes containing Parkinson disease-associated alpha-synuclein gene mutations precede central nervous system changes. Hum. Mol. Genet..

[93] Gispert S., Del Turco D., Garrett L., Chen A., Bernard D.J., Hamm-Clement J., Korf H.-W., Deller T., Braak H., Auburger G. (2003). Transgenic mice expressing mutant A53T human alpha-synuclein show neuronal dysfunction in the absence of aggregate formation. Mol. Cell. Neurosci..

[94] Pfeiffer R.F. (2011). Gastrointestinal dysfunction in Parkinson’s disease. Park. Relat. Disord..

[95] Noorian A.R., Rha J., Annerino D.M., Bernhard D., Taylor G.M., Greene J.G. (2012). Alpha-synuclein transgenic mice display age-related slowing of gastrointestinal motility associated with transgene expression in the vagal system. Neurobiol. Dis..

[96] Wang L., Fleming S.M., Chesselet M.-F., Taché Y. (2008). Abnormal colonic motility in mice overexpressing human wild-type alpha-synuclein. Neuroreport.

[97] Lam H.A., Wu N., Cely I., Kelly R.L., Hean S., Richter F., Magen I., Cepeda C., Ackerson L.C., Walwyn W. (2011). Elevated tonic extracellular dopamine concentration and altered dopamine modulation of synaptic activity precede dopamine loss in the striatum of mice overexpressing human α-synuclein. J. Neurosci. Res..

[98] Chesselet M.-F., Richter F. (2011). Modelling of Parkinson’s disease in mice. Lancet Neurol..

[99] Schaffernicht G., Shang Q., Stievenard A., Bötzel K., Dening Y., Kempe R., Toussaint M., Gündel D., Kranz M., Reichmann H. (2021). Pathophysiological Changes in the Enteric Nervous System of Rotenone-Exposed Mice as Early Radiological Markers for Parkinson’s Disease. Front. Neurol..

[100] Pan-Montojo F., Schwarz M., Winkler C., Arnhold M., O’Sullivan G.A., Pal A., Said J., Marsico G., Verbavatz J.-M., Rodrigo-Angulo M. (2012). Environmental toxins trigger PD-like progression via increased alpha-synuclein release from enteric neurons in mice. Sci. Rep..

[101] Klingelhoefer L., Reichmann H. (2015). Pathogenesis of Parkinson disease--the gut-brain axis and environmental factors. Nat. Rev. Neurol..

[102] Pan-Montojo F.J., Funk R.H.W. (2010). Oral administration of rotenone using a gavage and image analysis of alpha-synuclein inclusions in the enteric nervous system. J. Vis. Exp..

[103] Arnhold M., Dening Y., Chopin M., Arévalo E., Schwarz M., Reichmann H., Gille G., Funk R.H.W., Pan-Montojo F. (2016). Changes in the sympathetic innervation of the gut in rotenone treated mice as possible early biomarker for Parkinson’s disease. Clin. Auton. Res..

[104] Sharrad D.F., Chen B.N., Gai W.P., Vaikath N., El-Agnaf O.M., Brookes S.J.H. (2017). Rotenone and elevated extracellular potassium concentration induce cell-specific fibrillation of α-synuclein in axons of cholinergic enteric neurons in the guinea-pig ileum. Neurogastroenterol. Motil..

[105] Paillusson S., Tasselli M., Lebouvier T., Mahé M.M., Chevalier J., Biraud M., Cario-Toumaniantz C., Neunlist M., Derkinderen P. (2010). α-Synuclein expression is induced by depolarization and cyclic AMP in enteric neurons. J. Neurochem..

[106] Camilleri M., Cowen T., Koch T.R. (2008). Enteric neurodegeneration in ageing. Neurogastroenterol. Motil..

[107] Phillips R.J., Powley T.L. (2007). Innervation of the gastrointestinal tract: Patterns of aging. Auton. Neurosci..

[108] Phillips R.J., Walter G.C., Ringer B.E., Higgs K.M., Powley T.L. (2009). Alpha-synuclein immunopositive aggregates in the myenteric plexus of the aging Fischer 344 rat. Exp. Neurol..

[109] Braak H., Ghebremedhin E., Rüb U., Bratzke H., Del Tredici K. (2004). Stages in the development of Parkinson’s disease-related pathology. Cell Tissue Res..

[110] Wolters E.C., Braak H. (2006). Parkinson’s Disease: Premotor Clinico-Pathological Correlations. Parkinson’s Disease and Related Disorders.

[111] Krogh K., Christensen P. (2009). Neurogenic colorectal and pelvic floor dysfunction. Best Pract. Res. Clin. Gastroenterol..

[112] Marrinan S., Emmanuel A.V., Burn D.J. (2014). Delayed gastric emptying in Parkinson’s disease. Mov. Disord..

[113] Pfeiffer R.F. (2003). Gastrointestinal dysfunction in Parkinson’s disease. Lancet Neurol..

[114] Taguchi T., Ikuno M., Yamakado H., Takahashi R. (2020). Animal model for prodromal parkinson’s disease. Int. J. Mol. Sci..

[115] Liepelt-Scarfone I., Ophey A., Kalbe E. (2022). Cognition in prodromal Parkinson’s disease. Prog. Brain Res..

[116] Solla P., Wang Q., Frau C., Floris V., Loy F., Sechi L.A., Masala C. (2023). Olfactory impairment is the main predictor of higher scores at REM sleep behavior disorder (RBD) screening questionnaire in parkinson’s disease patients. Brain Sci..

[117] Erkkinen M.G., Kim M.-O., Geschwind M.D. (2018). Clinical neurology and epidemiology of the major neurodegenerative diseases. Cold Spring Harb. Perspect. Biol..

[118] Braak H., Del Tredici K. (2017). Neuropathological Staging of Brain Pathology in Sporadic Parkinson’s disease: Separating the Wheat from the Chaff. J. Park. Dis..

[119] Yilmaz R., Hopfner F., van Eimeren T., Berg D. (2019). Biomarkers of Parkinson’s disease: 20 years later. J. Neural Transm..

[120] Breit S., Kupferberg A., Rogler G., Hasler G. (2018). Vagus Nerve as Modulator of the Brain-Gut Axis in Psychiatric and Inflammatory Disorders. Front. Psychiatry.

[121] Liu B., Fang F., Pedersen N.L., Tillander A., Ludvigsson J.F., Ekbom A., Svenningsson P., Chen H., Wirdefeldt K. (2017). Vagotomy and Parkinson disease: A Swedish register-based matched-cohort study. Neurology.

[122] Kelly M.J., Breathnach C., Tracey K.J., Donnelly S.C. (2022). Manipulation of the inflammatory reflex as a therapeutic strategy. Cell Rep. Med..

[123] Parkkinen L., Pirttilä T., Alafuzoff I. (2008). Applicability of current staging/categorization of alpha-synuclein pathology and their clinical relevance. Acta Neuropathol..

[124] Frigerio R., Fujishiro H., Ahn T.-B., Josephs K.A., Maraganore D.M., DelleDonne A., Parisi J.E., Klos K.J., Boeve B.F., Dickson D.W. (2011). Incidental Lewy body disease: Do some cases represent a preclinical stage of dementia with Lewy bodies?. Neurobiol. Aging.

[125] Koga S., Sekiya H., Kondru N., Ross O.A., Dickson D.W. (2021). Neuropathology and molecular diagnosis of Synucleinopathies. Mol. Neurodegener..

[126] Macefield V.G., Henderson L.A. (2019). Identification of the human sympathetic connectome involved in blood pressure regulation. NeuroImage.

[127] Horsager J., Andersen K.B., Knudsen K., Skjærbæk C., Fedorova T.D., Okkels N., Schaeffer E., Bonkat S.K., Geday J., Otto M. (2020). Brain-first versus body-first Parkinson’s disease: A multimodal imaging case-control study. Brain.

[128] Socała K., Doboszewska U., Szopa A., Serefko A., Włodarczyk M., Zielińska A., Poleszak E., Fichna J., Wlaź P. (2021). The role of microbiota-gut-brain axis in neuropsychiatric and neurological disorders. Pharmacol. Res..

[129] Dash S., Syed Y.A., Khan M.R. (2022). Understanding the role of the gut microbiome in brain development and its association with neurodevelopmental psychiatric disorders. Front. Cell Dev. Biol..

[130] Baj A., Moro E., Bistoletti M., Orlandi V., Crema F., Giaroni C. (2019). Glutamatergic Signaling along the Microbiota-Gut-Brain Axis. Int. J. Mol. Sci..

[131] Parker A., Fonseca S., Carding S.R. (2020). Gut microbes and metabolites as modulators of blood-brain barrier integrity and brain health. Gut Microbes.

[132] Caspani G., Kennedy S., Foster J.A., Swann J. (2019). Gut microbial metabolites in depression: Understanding the biochemical mechanisms. Microb. Cell.

[133] Kim S., Kwon S.-H., Kam T.-I., Panicker N., Karuppagounder S.S., Lee S., Lee J.H., Kim W.R., Kook M., Foss C.A. (2019). Transneuronal Propagation of Pathologic α-Synuclein from the Gut to the Brain Models Parkinson’s Disease. Neuron.

[134] Wallen Z.D., Demirkan A., Twa G., Cohen G., Dean M.N., Standaert D.G., Sampson T.R., Payami H. (2022). Metagenomics of Parkinson’s disease implicates the gut microbiome in multiple disease mechanisms. Nat. Commun..

[135] Zhu M., Liu X., Ye Y., Yan X., Cheng Y., Zhao L., Chen F., Ling Z. (2022). Gut microbiota: A novel therapeutic target for parkinson’s disease. Front. Immunol..

[136] Chen S.-J., Lin C.-H. (2022). Gut microenvironmental changes as a potential trigger in Parkinson’s disease through the gut-brain axis. J. Biomed. Sci..

[137] Misera A., Łoniewski I., Palma J., Kulaszyńska M., Czarnecka W., Kaczmarczyk M., Liśkiewicz P., Samochowiec J., Skonieczna-Żydecka K. (2023). Clinical significance of microbiota changes under the influence of psychotropic drugs. An updated narrative review. Front. Microbiol..

[138] Horsager J., Knudsen K., Sommerauer M. (2022). Clinical and imaging evidence of brain-first and body-first Parkinson’s disease. Neurobiol. Dis..

[139] Molla M.D., Akalu Y., Geto Z., Dagnew B., Ayelign B., Shibabaw T. (2020). Role of Caspase-1 in the Pathogenesis of Inflammatory-Associated Chronic Noncommunicable Diseases. J. Inflamm. Res..

[140] Parada Venegas D., De la Fuente M.K., Landskron G., González M.J., Quera R., Dijkstra G., Harmsen H.J.M., Faber K.N., Hermoso M.A. (2019). Short Chain Fatty Acids (SCFAs)-Mediated Gut Epithelial and Immune Regulation and Its Relevance for Inflammatory Bowel Diseases. Front. Immunol..

[141] Pellegrini C., D’Antongiovanni V., Miraglia F., Rota L., Benvenuti L., Di Salvo C., Testa G., Capsoni S., Carta G., Antonioli L. (2022). Enteric α-synuclein impairs intestinal epithelial barrier through caspase-1-inflammasome signaling in Parkinson’s disease before brain pathology. npj Park. Dis..

[142] Muenter M.D., Tyce G.M. (1971). L-dopa therapy of Parkinson’s disease: Plasma L-dopa concentration, therapeutic response, and side effects. Mayo Clin. Proc..

[143] Poewe W., Antonini A. (2015). Novel formulations and modes of delivery of levodopa. Mov. Disord..

[144] Ouchi Y., Kanno T., Okada H., Yoshikawa E., Futatsubashi M., Nobezawa S., Torizuka T., Tanaka K. (2001). Changes in dopamine availability in the nigrostriatal and mesocortical dopaminergic systems by gait in Parkinson’s disease. Brain.

[145] Kouli A., Torsney K.M., Kuan W.-L., Stoker T.B., Greenland J.C. (2018). Parkinson’s disease: Etiology, neuropathology, and pathogenesis. Parkinson’s Disease: Pathogenesis and Clinical Aspects.

[146] Chen M., Mor D.E. (2023). Gut-to-Brain α-Synuclein Transmission in Parkinson’s Disease: Evidence for Prion-like Mechanisms. Int. J. Mol. Sci..

[147] Buhusi M., Olsen K., Yang B.Z., Buhusi C.V. (2016). Stress-Induced Executive Dysfunction in GDNF-Deficient Mice, A Mouse Model of Parkinsonism. Front. Behav. Neurosci..

[148] Aarsland D., Andersen K., Larsen J.P., Perry R., Wentzel-Larsen T., Lolk A., Kragh-Sørensen P. (2004). The rate of cognitive decline in Parkinson’s disease. Arch. Neurol..

[149] Clairembault T., Leclair-Visonneau L., Neunlist M., Derkinderen P. (2015). Enteric glial cells: New players in Parkinson’s disease?. Mov. Disord..

[150] Derkinderen P., Rouaud T., Lebouvier T., Bruley des Varannes S., Neunlist M., De Giorgio R. (2011). Parkinson disease: The enteric nervous system spills its guts. Neurology.

[151] Goldman J.G., Postuma R. (2014). Premotor and nonmotor features of Parkinson’s disease. Curr. Opin. Neurol..

[152] Del Rey N.L.-G., Quiroga-Varela A., Garbayo E., Carballo-Carbajal I., Fernández-Santiago R., Monje M.H.G., Trigo-Damas I., Blanco-Prieto M.J., Blesa J. (2018). Advances in parkinson’s disease: 200 years later. Front. Neuroanat..

[153] Simon D.K., Tanner C.M., Brundin P. (2020). Parkinson’s disease epidemiology, pathology, genetics, and pathophysiology. Clin. Geriatr. Med..

[154] Fasano A., Visanji N.P., Liu L.W.C., Lang A.E., Pfeiffer R.F. (2015). Gastrointestinal dysfunction in Parkinson’s disease. Lancet Neurol..

[155] Adler C.H., Beach T.G. (2016). Neuropathological basis of nonmotor manifestations of Parkinson’s disease. Mov. Disord..

[156] Alafuzoff I., Parkkinen L. (2014). Staged pathology in Parkinson’s disease. Park. Relat. Disord..

[157] Van Den Berge N., Ulusoy A. (2022). Animal models of brain-first and body-first Parkinson’s disease. Neurobiol. Dis..

